# Computational analysis of the effect of mitral and aortic regurgitation on the function of ventricular assist devices using 3D cardiac electromechanical model

**DOI:** 10.1007/s11517-017-1727-6

**Published:** 2017-10-28

**Authors:** Yoo Seok Kim, Ana R. Yuniarti, Kwang-Soup Song, Natalia A. Trayanova, Eun Bo Shim, Ki Moo Lim

**Affiliations:** 1 Department of IT Convergence Engineering, Kumoh National Institute of Technology, 61 Daehak-ro, Gumi, Gyeongbuk 39253 Republic of South Korea; 2 Department of Medical IT Convergence Engineering, Kumoh National Institute of Technology, Gumi, Republic of South Korea; 3 Department of Biomedical Engineering, Johns Hopkins University, Baltimore, MD USA; 4 Department of Mechanical & Biomedical Engineering, Kangwon National University, Chuncheon, Republic of South Korea

**Keywords:** Valvular insufficiency, Left ventricular assist device, Computational model cardiovascular system

## Abstract

Valvular insufficiency affects cardiac responses and the pumping efficacy of left ventricular assist devices (LVADs) when patients undergo LVAD therapy. Knowledge of the effect of valvular regurgitation on the function of LVADs is important when treating heart failure patients. The goal of this study was to examine the effect of valvular regurgitation on the ventricular mechanics of a heart under LVAD treatment and the pumping efficacy of an LVAD using a computational model of the cardiovascular system. For this purpose, a 3D electromechanical model of failing ventricles in a human heart was coupled with a lumped-parameter model of valvular regurgitation and an LVAD-implanted vascular system. We used the computational model to predict cardiac responses with respect to the severity of valvular regurgitation in the presence of LVAD treatment. An LVAD could reduce left ventricle (LV) pressure (up to 34%) and end-diastolic ventricular volume (up to 80%) and maintain cardiac output at the estimated flow rate from the LVAD under the condition of mitral regurgitation (MR); however, the opposite would occur under the condition of aortic regurgitation (AR). Considering these physiological responses, we conclude that AR, and not MR, diminishes the pumping function of LVADs.

## Introduction

Left ventricular assist devices (LVADs) are mechanical devices that assist the function of a failed heart by facilitating blood circulation in the body. Recently, LVADs have been used as a bridge to transplantation [1, 2] or as a destination therapy [3]. Thus, it is important to improve coronary blood perfusion and reduce ventricular afterload as well as maintain normal cardiac output to the whole body [4, 5].

Valvular regurgitation is a disorder of the heart in which the valve does not close properly while the heart is pumping in or pumping out blood. The most common heart valve diseases are aortic and mitral insufficiency. Depending on the valve involved and the severity of regurgitation, cardiac responses, such as cardiac output, blood pressure, and stroke work, vary appreciably [6, 7]. Furthermore, regurgitation can also affect the pumping efficacy in LVAD therapy [3]. Several studies have focused on determining regurgitation volume using medical imaging techniques [2, 8–11] and on predicting the effect of regurgitant volume on ventricular mechanical function [12]. However, the effect of a specific valvular regurgitation on the function of an LVAD in the failing ventricles of patients treated with LVAD therapy has not been studied. Although experiments that predict the pumping efficacy of an LVAD for different degrees of valvular insufficiency can be performed, measurement of cardiac responses such as the ventricular unloading effect of an LVAD is hampered by low spatiotemporal resolution.

Computational studies offer an attractive alternative to the experimental studies. Currently, a computational model of the heart (i.e., cardiac electromechanical model) enables us to present a multiscale framework, from cell to tissue and from electrophysiology to organ mechanics, to simulate the function of the entire heart. Specifically, the electromechanical model is capable of capturing the effect of electrical activity that induces cardiac mechanics through excitation-contraction coupling (ECC), or conversely, the effect of mechanical events induces the alteration of electrical activity through mechanoelectrical feedback (MEF) [13, 14]. With the recent advances in high-performance computing, the finite element-based electromechanical model of the heart [9, 15–22] can be coupled with the circulatory system [23–26] to represent how the cellular model triggers the electrical waves and cardiac contraction, as well as the hemodynamic responses due to blood flow mechanics. We have published several studies in which we used this computational simulation to compare the effects of ventricular unloading for different types of pumping [19, 20] and cannulation sites [21] and the effect of valvular regurgitation on cardiac mechanics [6].

The goal of this study was to examine the effect of valvular regurgitation on the ventricular mechanics in a heart under LVAD and the pumping efficacy of an LVAD using a computational model of the cardiovascular system. We coupled the 3D electromechanical model of failing ventricles in a human heart, used in our previous study [17] with a lumped-parameter model of valvular regurgitation and an LVAD-implanted vascular system.

## Methods

### Model of human ventricles

**Fig. 1:**
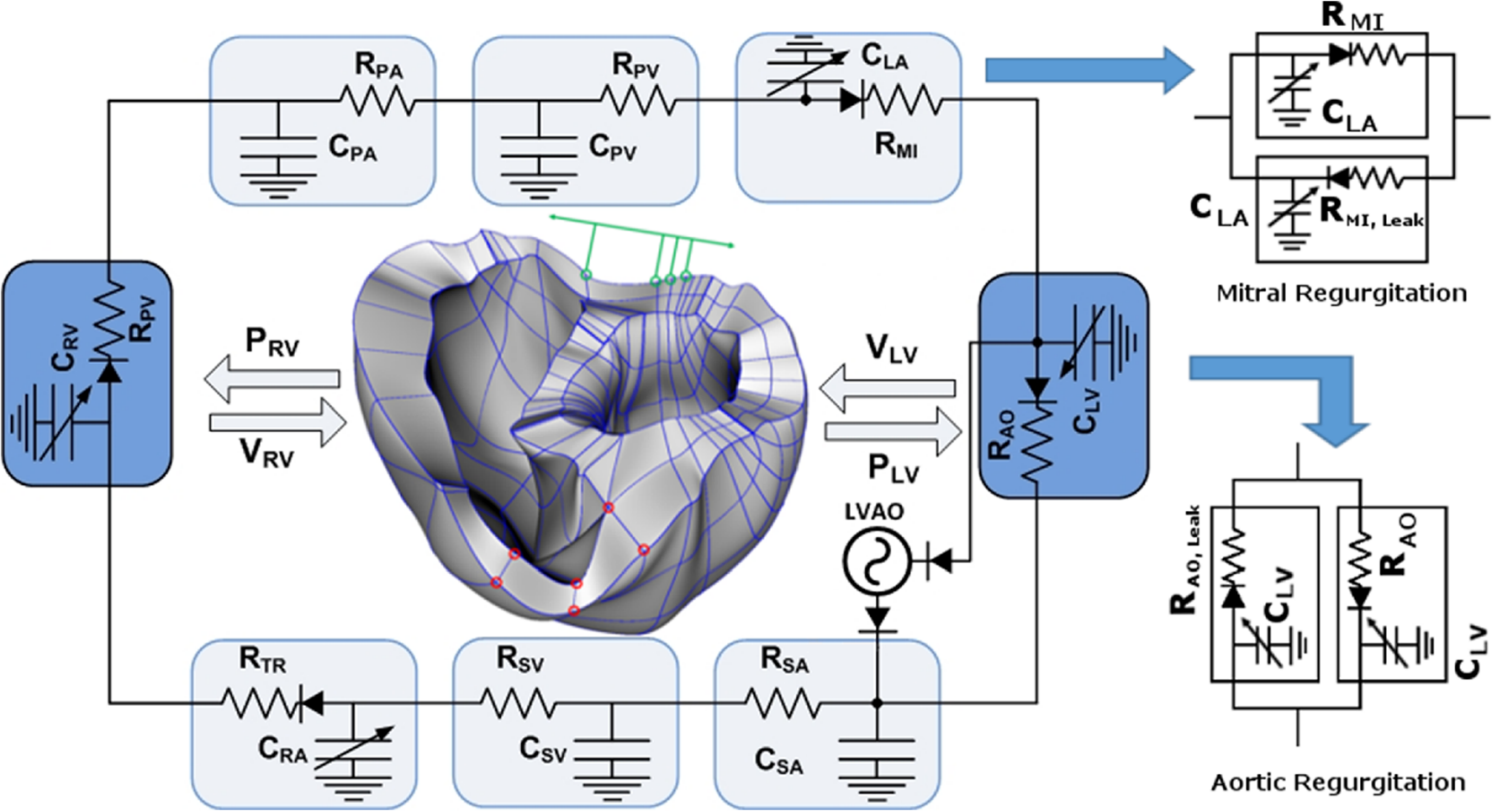
Schematic diagram of the finite element-based ventricular electromechanical model coupled with the circulatory and LVAD models. The human ventricular mechanical mesh with locations at which boundary conditions were applied is depicted in the center. *P*_*RV*_ RV pressure, *V*_*RV*_ RV volume, *P*_*LV*_ LV pressure, *V*_*LV*_ LV volume, *R*_*PA*_ pulmonary artery resistance, *C*_*PA*_ pulmonary artery compliance, *R*_*PV*_ pulmonary vein resistance, *C*_*PV*_ pulmonary vein compliance, *R*_*MI*_ mitral valve resistance, *C*_*LA*_ left atrium compliance, *R*_*AO*_ aortic valve resistance, *R*_*SA*_ systemic artery resistance, *C*_*SA*_ systemic artery compliance, *R*_*SV*_ systemic vein resistance, *C*_*SV*_ systemic vein compliance, *R*_*TR*_ tricuspid valve resistance, *C*_*RA*_ right atrium compliance, and *R*_*PU*_ pulmonary valve resistance, *red nodes* fixed-position boundary condition, *green nodes and arrows* boundary condition of restricting the movement to the direction indicated by the *green arrow*

To achieve the goals of this study, we used a previously developed magnetic resonance imaging (MRI)-based electromechanical model of failing ventricles in a human heart [17] coupled with a lumped-parameter model of the circulatory system (see Fig. 1). The ventricular geometry, fiber, and laminar sheet architecture of the model were constructed from high-resolution MRI and diffusion tensor MRI scans of human ventricles in the condition of heart failure (HF).

The electromechanical model is composed of two parts—an electrical component and a mechanical component—coupled via an intracellular calcium (Ca) transient, which links biophysically detailed models of local membrane kinetics and local cardiac myofilament dynamics throughout the ventricles. The myofilament model describes the binding of Ca to troponin C and the development of active tension. Deformation of the ventricles results from the active tension generated by the cardiac cells. Physiologically, as an electrical wave propagates through the heart, depolarization of each myocyte initiates release of Ca from intracellular stores. This is followed by the binding of Ca to troponin C and cross-bridge cycling, where the latter forms the basis of contractile protein movement and the development of active tension in the cell, resulting in deformation of the ventricles.

The electrical component of the model simulates the propagation of a wave of transmembrane potential by solving the monodomain equation on the electrical mesh. This equation describes the current flow through cardiac cells that are connected electrically to represent them as a continuum. The flow of current in the tissue is driven by active ion exchange across myocyte membranes. These processes are represented by the human ionic model of Tusscher et al. [27]. To account for the remodeling of the passive electrical properties associated with heart failure, the electrical conductivities were reduced by 30%, allowing for a total electrical activation time of 150 ms, according to the experimental results obtained by Helm et al. [28]. The simultaneous solution of the partial differential equation (PDE) for the electrical conduction model and the set of ordinary differential equations (ODEs) for the ionic model represent simulated electrical wave propagation in the heart.

Distributions of the Ca transient are obtained from the electrical component of the model. The Ca transient serves as input to the cell myofilament model that represents the generation of active tension within each myocyte and for which a set of ODEs and algebraic equations describes Ca binding to troponin C, cooperativity between regulatory proteins, and cross-bridge cycling. In this study, the peak of the obtained Ca transient was reduced to 70% of its value to introduce systolic dysfunction [29]. Ventricular contraction resulting from the generation of active tension was represented by the model of myofilament dynamics proposed by Rice et al. [30]. Ventricular deformation is described by the equations of passive cardiac mechanics, with the myocardium assumed an orthotropic, incompressible, and hyperelastic material with passive properties defined by an exponential strain energy function [21, 31]. Previous studies [17, 22, 28] chose to use the exponential strain energy function instead of the pole-zero law [18] because of its relative simplicity in estimating the parameters and its numerical stability. The passive scaling constant of the strain energy function was increased fivefold over the normal value to represent the increased stiffness of the failing myocardium [32]. A similar approach was used by others [6, 17, 33]. The simultaneous solution of the myofilament model equations with those representing passive cardiac mechanics on the mechanical mesh constitutes the simulation of cardiac contraction.

To determine the appropriate boundary conditions for ventricular deformation, we analyzed the open-access animation frames of cine MRI (INRIA, Asclepios Research Project, Sophia Antipolis Cedex, France). According to the animation, the portion of the ventricle near to the aperture of the pulmonary artery in the right ventricle remained static during contraction. In addition, the movement of the surface of the posterior wall near the septum was found to be limited to a single direction in the short-axis plane, tangential to the surface of the ventricles. These boundary conditions were then applied in the model by fixing the positions of the red nodes in Fig. 1 and restricting the movement of the green nodes to the direction indicated by the green arrow (see the human ventricle mesh located in the center of Fig. 1). In addition, the movement of the entire ventricular base was restricted to a fixed plane perpendicular to the longitudinal axis of the ventricles, similar to a previous study [22].

### Model of LVAD function

As in our previous study [19], we combined the electromechanical model of failing ventricles with the lumped-parameter model of the circulatory system and the LVAD pump model. The LVAD component was connected to the ventricular and circulatory models through the inlet in the LV, and the outlet was inserted into the aorta in the circulatory model. Briefly, the LVAD component was modeled as a flow generator with a specific mean flow rate of 40 mL/s. A constant flow condition was used to simulate a continuous LVAD.

### Model of valvular regurgitation

To model mitral regurgitation (MR) and aortic regurgitation (AR), two branches were added to the aortic and mitral compartments in the lumped-parameter model (Fig. 1). One branch had a forward diode to represent forward flow, and the other branch had a backward diode to represent leakage flow; the diodes had different resistance values. Regurgitant flow dynamics through the mitral and aortic valves is defined by the following equations:

1 $$ {Q}_{\mathrm{MI}}=\left\{\begin{array}{l}\frac{P_{\mathrm{LA}}-{P}_{\mathrm{LV}}}{R_{\mathrm{MI}}}\kern8.25em ,\mathrm{when}\ {P}_{\mathrm{LA}}>{P}_{\mathrm{LV}}\ \\ {}\frac{P_{\mathrm{LA}}-{P}_{\mathrm{LV}}}{R_{\mathrm{MI},\mathrm{Leak}}}=\frac{P_{\mathrm{LA}}-{P}_{\mathrm{LV}}}{R_{\mathrm{MI}}}\times \frac{\mathrm{SF}}{100}\kern1.25em ,\mathrm{when}\ {P}_{\mathrm{LA}}\le {P}_{\mathrm{LV}}\end{array}\right. $$

2 $$ \left\{\begin{array}{l}\frac{P_{\mathrm{LV}}-{P}_{\mathrm{AO}}}{R_{\mathrm{AO}}}\kern8.25em ,\mathrm{when}\ {P}_{\mathrm{LV}}>{P}_{\mathrm{AO}}\ \\ {}\frac{P_{\mathrm{LV}}-{P}_{\mathrm{AO}}}{R_{\mathrm{AO},\mathrm{Leak}}}=\frac{P_{\mathrm{LV}}-{P}_{\mathrm{AO}}}{R_{\mathrm{AO}}}\times \frac{\mathrm{SF}}{100}\kern1.25em ,\mathrm{when}\ {P}_{\mathrm{LV}}\le {P}_{\mathrm{AO}}\end{array}\right. $$

where *Q*, *P*, and *R* denote the flow rate (mL/min), pressure (mmHg), and flow resistance (mmHg min/mL), respectively; the subscripts MI, AO, LV, LA, and Leak represent the mitral valve, aortic valve, left ventricle, left atrium, and leakage, respectively; SF represents a scale factor for regurgitation severity, which varies from 0 to 10%.

### Simulation protocol

Ventricular contraction of a failing heart was simulated with and without LVAD support. For all simulations, the entire cardiac cycle lasted 600 ms. The severity of regurgitation was changed from 0% (baseline state) to 10% (severe regurgitation) in 2% increments. For each case, the simulation was executed for 20 s to ensure that cardiovascular responses such as blood pressure, flow, and volume reached a nearly steady state in each compartment. To analyze the effect of regurgitation on ventricular wall mechanics, we computed the changes in ventricular strain, stroke work, regurgitant volume, and regurgitant fraction for the different severity levels of regurgitation.

## Results

**Fig. 2:**
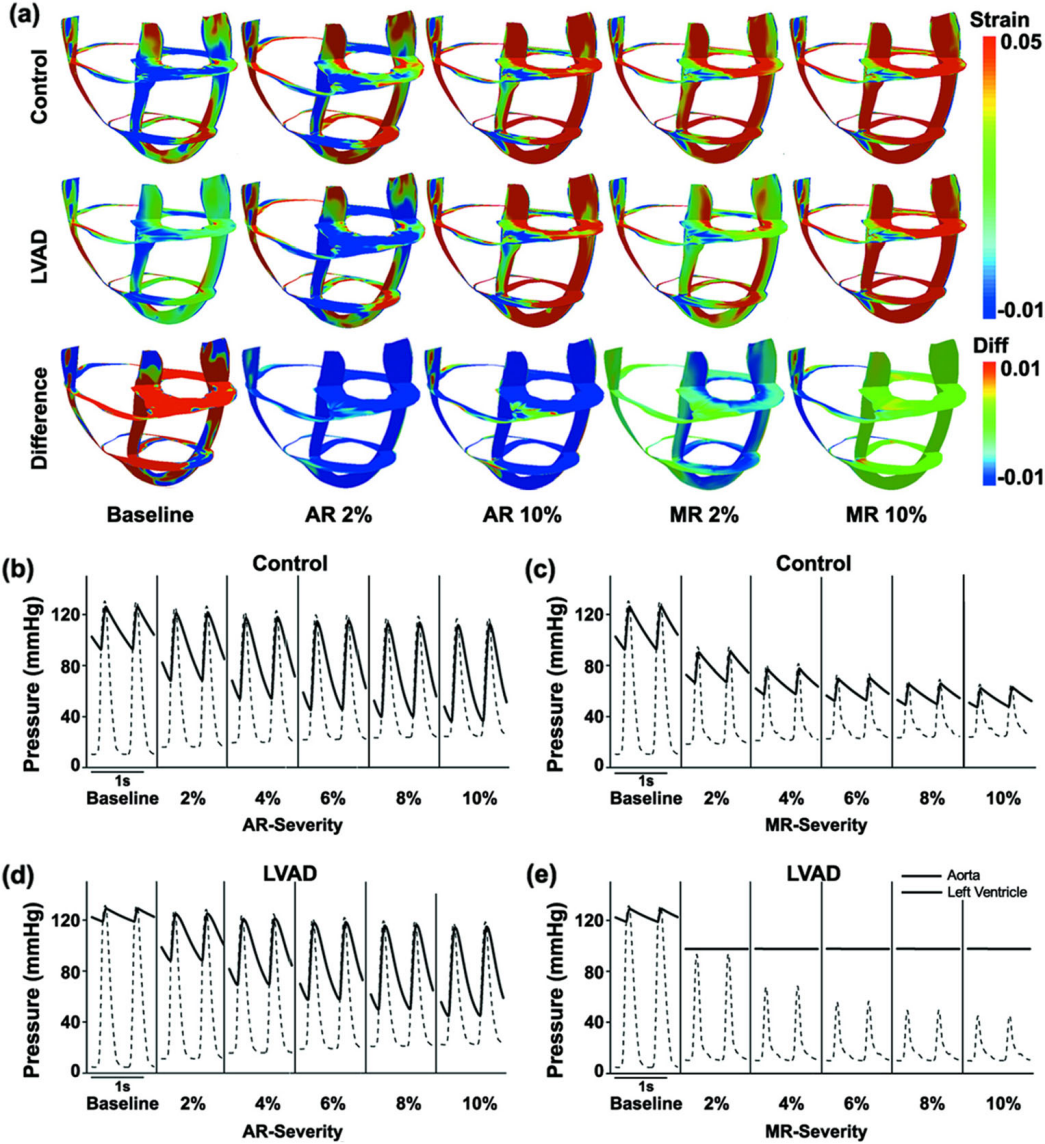
**a** Transmural distribution of myocardial strain in HF ventricles under baseline, AR, and MR conditions without LVAD support (*Control*), with continuous LVAD support (*LVAD*), and the difference between the two (*Difference*). First column (*Baseline*) shows HF without regurgitation; second (AR 2%) and third (AR 10%) columns show 2 and 10% AR conditions, respectively; and fourth (MR 2%) and fifth (MR 10%) columns show 2 and 10% MR conditions, respectively. Pressure waveforms of LV and aorta for **b** AR and **c** MR without LVAD support and **d** AR and **e** MR with LVAD support

The transmural distribution of myocardial strain in failed ventricles is shown in Fig. 2a for baseline (first column), AR (second and third columns), and MR (fourth and fifth columns). The first row shows myocardial strain in the control group, which was not treated by LVAD; the second row shows myocardial strain in the LVAD therapy group; and the third row shows the difference between the control and LVAD therapy groups. In addition, the pressure waveforms of the left ventricle (LV) and the aorta for AR and MR in the control group (Fig. 2b and c, respectively) and AR and MR with LVAD support (Fig. 2d and e, respectively) are shown.

In the control group, the strain distribution slightly increased with the severity of AR 2% and increased more with the severity of AR 10%, MR 2%, and MR 10%, respectively (see Fig. 2a). LV and aortic pressure decreased more under the MR condition than under the AR condition (see Fig. 2b, c). However, the pulse pressure of the aorta was greater under the AR condition than under the MR condition.

In the LVAD therapy group, myocardial strain was significantly decreased under the baseline condition in which there is no valvular insufficiency. However, myocardial strain was not markedly affected by LVAD treatment under the AR condition, although it was reduced significantly with LVAD treatment under the MR condition (see Fig. 2a). LV pressure decreased during diastole and slightly increased during systole for all LVAD therapy cases, whereas the aortic pressure increased under baseline and AR conditions and was maintained at 97.4 mmHg under the MR condition. This value was lower than that under the baseline condition, i.e., 131.36 mmHg.

**Fig. 3:**
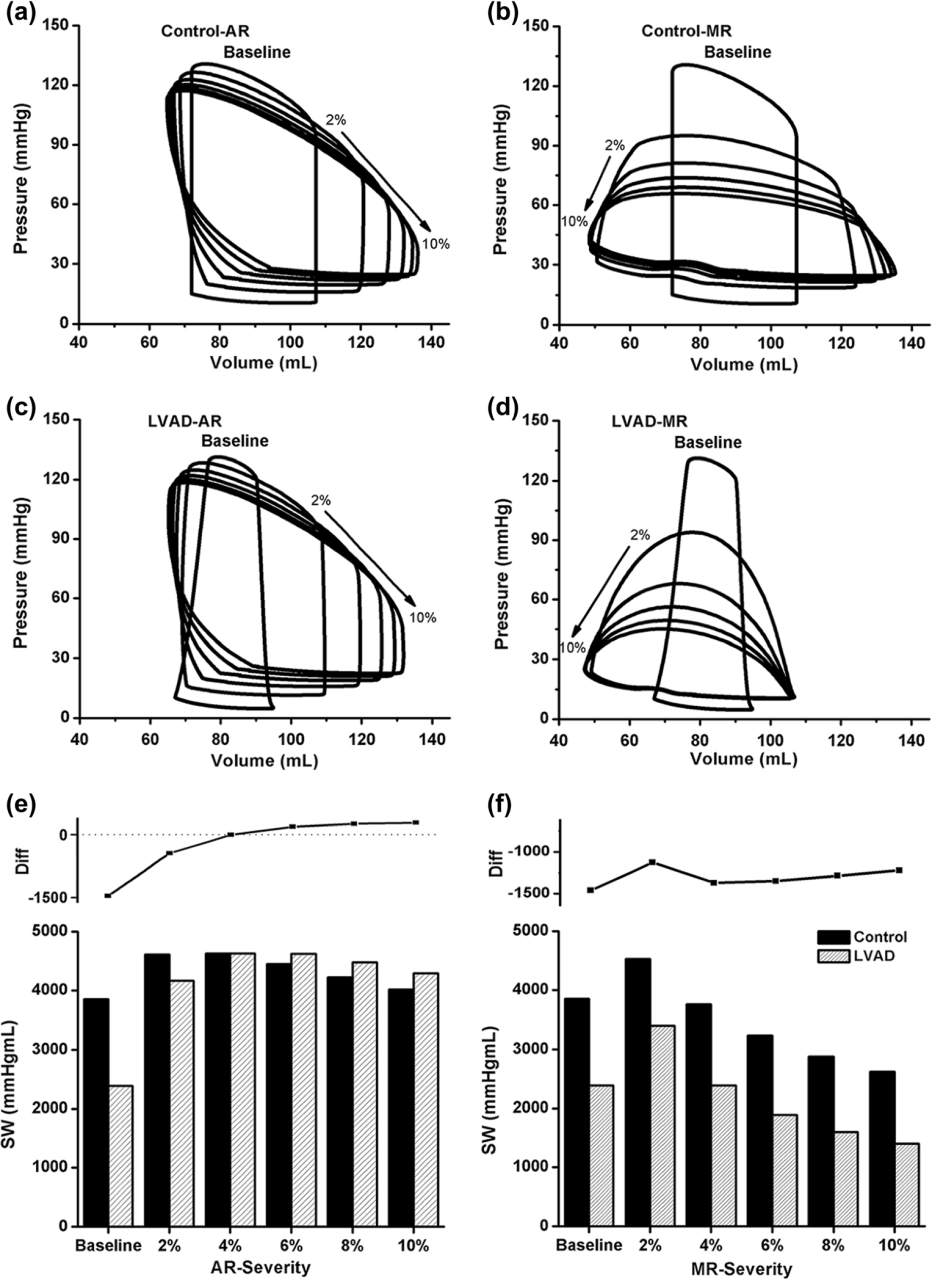
Pressure-volume curves for **a** AR ventricle, **b** MR ventricle without LVAD support, **c** AR ventricle, and **d** MR ventricle with LVAD support. Comparison of LVSW between the control and LVAD groups under various **e** AR and **f** MR conditions

Using the simulated data, we constructed LV pressure-volume curves for the control group (Fig. 3a, b) and the LVAD therapy group (Fig. 3c, d) under AR and MR conditions. By integrating the area in each pressure-volume loop, the LV stroke work (LVSW) was obtained and compared between the control and LVAD therapy groups under AR (Fig. 3e) and MR (Fig. 3f) conditions.

In the control group, when the severity of regurgitation increased, the end-diastolic volume increased (up to 130% under AR and 125% under MR) and the end-systolic volume decreased (up to 90% under AR and 68% under MR). Furthermore, LV systolic pressure decreased (up to 90% under AR and 51% under MR) and diastolic pressure increased (up to 200% under AR and 225% under MR) as a function of the severity of regurgitation (Fig. 3a, b). The shapes of the pressure-volume loops in the LVAD therapy group were similar to those in the control group under the AR condition (see Fig. 3a, c). In the control group, the LVSW increased up to a 2% severity of AR and MR and decreased thereafter; however, with LVAD treatment, the LVSW decreased under the baseline condition (energy unloading effect), a result we found in our previous study [25]. Similar to the baseline condition, LVSW decreased with LVAD treatment under the MR condition (Fig. 3f). However, LVSW increased with LVAD treatment when the severity of AR was 6, 8, and 10%, decreased when severity of AR was 2%, and stayed the same when the severity of AR was 4% (Fig. 3e).

**Fig. 4:**
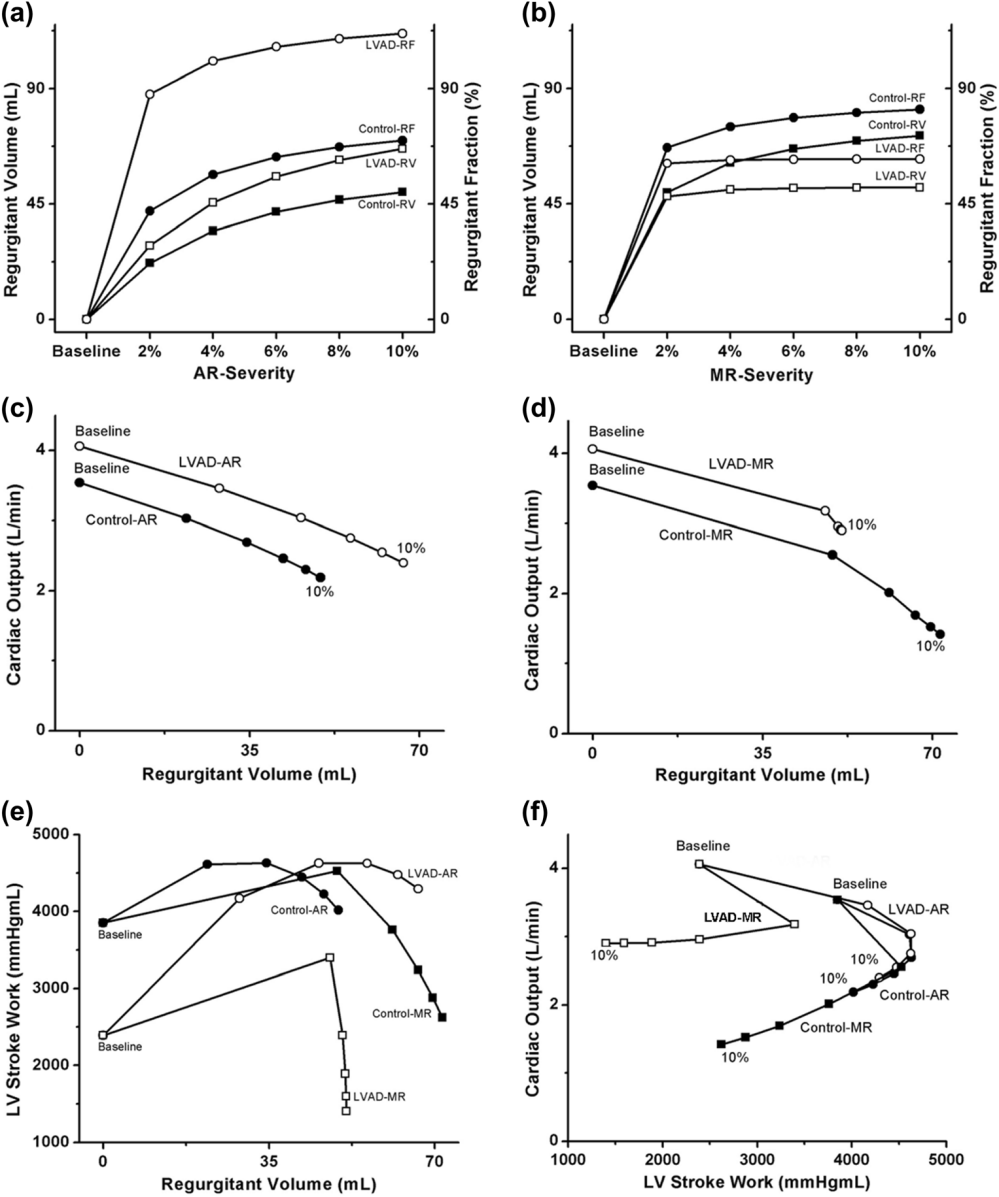
Regurgitant volume and fraction of the control and LVAD therapy groups under various **a** AR and **b** MR severity conditions. Cardiac output of the control and LVAD therapy groups under various **c** AR and **d** MR severity conditions. **e** LVSW of the control and LVAD therapy groups under various AR and MR severity conditions as a function of RV. **f** Cardiac output of the control and LVAD therapy groups under various AR and MR severity conditions as a function of the LVSW. *LV* left ventricle, *LVSW* left ventricle stroke work, *RV* regurgitant volume, *RF* regurgitant fraction, *Baseline* heart failure without valve regurgitation, *AR* aortic regurgitation, *MR* mitral regurgitation, *control* no LVAD support

Figure 4 shows the regurgitant volume (RV), regurgitation fraction (RF), cardiac output, LVSW, and the relationships between these parameters. RF is the percentage of blood that regurgitates back through the aortic valve to the LV because of aortic insufficiency or through the mitral valve to the atrium because of mitral insufficiency. It is the amount of blood regurgitated into a cardiac chamber divided by the stroke volume. RV and RF increased sharply with low levels of regurgitant severity but increased commensurately with increasing severity of AR and MR (Fig. 4a, b). Under the AR condition, LVAD treatment increased RF (significantly) and RV because of the increased forward flow into the aorta from LVAD pumping. Under the MR condition, LVAD reduced RV and RF.

Cardiac output decreased as a function of RV under both AR and MR conditions. When LVAD treatment was applied, cardiac output increased (530 mL/min for AR and 490 mL/min for MR in average), albeit to a lesser extent than the 4 L/min achieved under both AR and MR conditions (Fig. 4c, d). LVSW as a function of RV is shown in Fig. 4e. The plot of cardiac output as a function of LVSW (Fig. 4f), which indicates the energetic efficiency of blood pumping, was generated by combining Fig. 4d, e. Under identical stroke work conditions, cardiac output was greater in the LVAD therapy group (i.e., the LVAD improved blood circulation even under regurgitant conditions). Interestingly, the relationship between cardiac output and LVSW shifted up (i.e., cardiac output improved) and to the left (i.e., less stroke work) under the MR condition when the LVAD was implanted. In the control group, cardiac output did not linearly correlate with LVSW but decreased according to regurgitant severity. LVAD treatment increased cardiac output under both AR and MR conditions. Under MR condition, cardiac output decreased and stayed around 3 L/min (i.e., estimated flow rate of LVAD pumping) with respect to LVSW, which decreased according to MR severity. However, under the AR condition, cardiac output was not maintained at the level of the LVAD pumping rate but decreased according to AR severity. Furthermore, the LVSW continued to decrease according to AR severity, although LVSW increased for AR 4%.

## Discussion

The blood volume regurgitated from the aorta into the LV during diastole and from the LV into the LA during systole increases when the severity of AR and MR increases. This reduces cardiac output, the direction of which is from the LA through the LV and into the aorta. Therefore, more blood accumulates in the ventricles, which increases their end-diastolic volume (Fig. 3a, b). Under the AR condition, the aortic blood volume, which is pumped from the LV during systole, can flow back into the LV during diastole. Under the AR condition, the LV has two inlets, i.e., the mitral and aortic valves, but only one outlet, i.e., the aortic valve. This results in an increase in the LV diastolic volume, but there is a decrease in the LV systolic volume owing to the Frank Starling law (preload-dependent contractility) (Fig. 3a). Under the MR condition, LV blood is regurgitated into the LA cavity during ventricular systole, in addition to the typical blood flow into the LA through the pulmonary veins. Therefore, more blood flows from the LA into the LV during diastole, resulting in a larger end-diastolic volume for the LV. Under the MR condition, the LV has two outlets, i.e., the aortic and mitral valves, but only one inlet, i.e., the mitral valve. This results in a decrease in LV systolic pressure and volume (lower than that under the AR condition) owing to the small flow resistance in the two LV outflow tracts (Fig. 3b). Subsequently, there is reduced mechanical load on the ventricle because of the decrease in the myocardial strain (Fig. 2).

The LVAD is cannulated from the LV to the aorta so that it pumps blood from the LV into the aorta. Under the baseline condition, the LVAD increases cardiac output (see Fig. 4c or d) and aortic blood pressure to normal levels (Fig. 2d or e) and reduces ventricular pressure (Fig. 2d or e) and myocardial strain (Fig. 2a). Under the AR condition, nearly all of the blood pumped to the aorta by the LVAD flows back into the LV cavity. Therefore, all the advantages of the LVAD function are diminished. However, under the MR condition, the regurgitant blood volume to the LA decreases because the LVAD pumps blood from the LV into the aorta continuously. Therefore, MR affects the LVAD function to a lesser extent than AR.

Several physiological factors such as stroke work, cardiac output, and blood pressure can be used to estimate cardiac performance. In this study, cardiac output was used to quantify the practical performance of the ventricles and the LVAD because the main role of ventricular contraction and LVAD is an appropriate cardiac output. Stroke work is the work performed by the ventricles to develop the appropriate cardiac output. Cardiac output versus stroke work can be indicative of good physiological factors for estimating the work efficiency of LV. From these physiological factors, we conclude that AR, but not MR, perturbs the pumping function of the LVAD.

The present study has several limitations. First, no experimental or clinical data were obtained in the study. Instead, we used a validated cell model [27, 30] and methodologies [17, 19, 20, 26] from previous studies. For the cardiac electrophysiological model, we used the human ventricular cell model of Tusscher et al. [27], which had been validated with experimentally measured data. For the cardiac mechanical model, we used the myofilament dynamics model of Rice et al. [30]. Next, we implemented one-way ECC by triggering cross-bridge cycling with Ca dynamics but ignored the mechanoelectrical feedback mechanism. To avoid making the model too complex, we did not include a coronary circulation model but did use a lumped-parameter representation of valvular regurgitation. These limitations are not expected to significantly alter the main findings of the study.

## Conclusion

We compared the effects of MR and AR on the LVAD pumping function using a sophisticated computational model of the cardiovascular system (Fig. 1). Six scenarios were compared in the computational experiments: the control-baseline group (no regurgitation under no LVAD support), control-AR group (AR but no LVAD support), control-MR group (MR but no LVAD support), LVAD-baseline group (no regurgitation under LVAD support), LVAD-AR group (AR under LVAD support), and LVAD-MR group (MR under LVAD support). The major roles of the LVAD are to reduce the mechanical load on the ventricle and maintain cardiac output at a normal level. The LVAD pumping function was estimated by predicted cardiovascular responses of the LVAD-implanted cardiovascular system model, including the myocardial strain, LV and aortic blood pressure, LV pressure-volume curves, LVSW, and cardiac output. The main findings were as follows:

1. The LVAD reduced myocardial strain and LV pressure and maintained constant arterial pressure of 97.4 mmHg under the MR condition. However, it did not markedly reduce strain and LV pressure under the AR condition (see Fig. 2).
2. The LVAD reduced LV end-diastolic volume (volume unloading) under the MR condition but not under the AR condition (see Fig. 3).
3. The LVAD maintained cardiac output at the estimated flow rate (i.e., 3 L/min) from the blood pump under the MR condition; however, cardiac output was not maintained at the estimated flow rate; hence, it could not be estimated (see Fig. 4).
